# Perceptions of swine euthanasia among Brazilian caretakers from non-integrated swine farms

**DOI:** 10.3389/fvets.2024.1513141

**Published:** 2025-01-06

**Authors:** Laya Kannan Silva Alves, Monique Danielle Pairis-Garcia, Andréia Gonçalves Arruda, Cecília Archangelo Ferreira de Melo, Nadia de Almeida Ciriaco Gomes, Roberta Yukari Hoshino, Cesar Augusto Pospissil Garbossa

**Affiliations:** ^1^Laboratory of Swine Research, Department of Nutrition and Animal Production, School of Veterinary Medicine and Animal Science, University of São Paulo, Pirassununga, SP, Brazil; ^2^Global Production Animal Welfare Laboratory, Department of Population Health and Pathobiology, College of Veterinary Medicine, North Carolina State University, Raleigh, NC, United States; ^3^Department of Veterinary Preventive Medicine, College of Veterinary Medicine, The Ohio State University, Columbus, OH, United States

**Keywords:** attitudes, decision-making, on-farm euthanasia, pigs, timely euthanasia, training, welfare

## Abstract

Timely and humane euthanasia is crucial for animal welfare on swine farms, yet challenges persist in its implementation, particularly in Brazil, where the responsibility often falls to caretakers lacking training. This study aimed to assess the knowledge, attitudes, and practices of swine caretakers regarding euthanasia across non-integrated farms (ranging from 1,000 to 3,500 housed sows) and different experience levels (from less than a month to 40 years working with pigs). A total of 117 people directly working with pigs participated in a survey designed to evaluate their decision-making skills, euthanasia competencies, and understanding of Brazilian guidelines for euthanasia methods. Using Cluster analysis, we identified two distinct groups of caretakers: (1) Empathetic, self-sufficient, apathetic about euthanasia; and (2) Empathetic, knowledge seeker, uncomfortable with euthanasia. Both Clusters exhibited high empathy toward pigs and confidence in identifying sick animals but differed in their attitudes toward euthanasia. The risk factor analysis showed a tendency for younger respondents (under 36 years old) and those from smaller farms (less than 2,000 sows) were more likely to belong to Cluster 2, while older caretakers (over 36 years) and those working on larger farms (more than 2,000 housed sows) tended to belong to Cluster 1. Furthermore, a significant proportion of caretakers lacked knowledge of the euthanasia Brazilian guidelines, as evidenced by incorrect responses regarding acceptable euthanasia methods, such as performing cardiac perforation or using non-penetrating captive bolt guns on growing-finishing pigs. This study highlights the variability in caretaker experience and attitudes toward euthanasia, suggesting a critical need for targeted training programs and euthanasia protocols that address both emotional and practical aspects. Improved understanding of caretaker attitudes can enhance both human and animal welfare on farms.

## 1 Introduction

Timely and humane euthanasia is a subject of global animal welfare significance that has been extensively studied, particularly in food animal medicine, to improve the lives of farm animals by minimizing pain and suffering experienced at the end of life (1, 2). The term euthanasia originates from the Greek words “*eu”* and *“thanatos*,” meaning “good death.” It represents a critical component of on-farm management, primarily implemented to alleviate the suffering of diseased or injured animals with little to no chance of recovery (3). Despite its acceptance as a humane tool, determining the appropriate time to euthanize remains challenging (4).

In Brazil, euthanasia is classified as a necessary clinical procedure authorized solely for veterinarians [Resolution No 1000, (5)], with guidelines and training materials provided by the Federal Veterinary Medical Council (6) and the Ministry of Agriculture, Livestock and Food Supply (7). However, Brazil’s status as a key contributor of swine industry, producing over 40 million pigs annually and ranking 4th in global production (8), presents unique challenges for ensuring humane euthanasia across its expansive swine industry. The vast inventory of pigs, spread across all 26 states (9), make it impractical to rely on veterinarians as the sole source for conducting euthanasia when needed (10). As a result, most on-farm euthanasia specific to swine is performed by caretakers responsible for daily animal care (11).

Caretakers have not historically received formal training related to the euthanasia decision-making process or the implementation of the procedure itself. In fact, recent work published in 2019 conducted surveys on 371 caretakers working on integrated swine farms in the South of Brazil assessing knowledge and training specific to euthanasia. Only 7% of individuals participating in the study had received any training regarding on-farm euthanasia, and over 90% of surveyed swine caretakers were uncomfortable with making euthanasia decisions and performing the procedure (10). The decision-making process for euthanasia is complex and often influenced by several variables that may impact the caretaker’s ability to perform euthanasia. These variables include, but are not limited to, access to formal training opportunities to perform euthanasia, written standards operating procedures outlining the euthanasia process, appropriate and functioning euthanasia equipment, and time during working hours to perform euthanasia in addition to other job tasks (12). In addition, caretaker’s previous experience with euthanasia and emotional attachment towards the animal can also impact their ability to humanely perform euthanasia in a timely manner (13).

Identifying barriers to conducting timely euthanasia is crucial for ensuring on-farm animal welfare, particularly given that those responsible for performing euthanasia have not been trained and are not comfortable performing the task. Therefore, the objective of this study was to assess Brazilian caretakers’ knowledge and attitudes towards on-farm swine euthanasia and its association to individual’s demographic characteristics, with the goal of identifying barriers to implementing euthanasia in a timely manner.

## 2 Materials and methods

The study was conducted according to the guidelines of the Institutional Research Ethics Committee for Human Subjects (CEPH) of the Faculty of Animal Science and Food Engineering (FZEA) at the University of São Paulo (USP; #5.674.052).

### 2.1 Survey

Non-integrated farrow-to-finish swine farms located in São Paulo and Minas Gerais States, Brazil, were recruited through investigators professional network via direct phone calls and emails to participate in this study. Farms that expressed interest in participating worked directly with the authors to plan and schedule visits. Over a six-month period (January to June 2023), two of the authors (LKSA, NACG) visited the participating farms multiple times as part of a larger project, during which data was continuously collected. The researchers stayed on the farms for 1 week per visit to ensure that all caretakers had an opportunity to participate. Surveys were administered during the caretaker’s free time throughout the day. During these visits, any individual currently working on the farm with direct responsibility for animal care was invited to participate. Prior to obtaining access to the survey, participants were required to sign a consent form authorizing the use of their anonymous responses and demographic information by the researchers. Upon signing the consent form, participants had access to the written survey, which was provided in Portuguese.

The survey consisted of three sections, with a total of 78 questions. The survey questions were adapted from previous frameworks developed by Rault et al. (14, swine) and Merenda et al. (12, dairy) and included questions to assess caretakers’ characteristics, attitudes towards euthanasia, and factors influencing the decision-making process (e.g., inadequate knowledge, knowledge-seeking, and self-assessed confidence).

The first section of the survey, available in Appendix 1, collected demographic information, through multiple-choice questions, including residence, age, sex, race, and ethnicity, experience with livestock animals, role on the farm, farm size, previous euthanasia experience, and euthanasia training.

The second section of the survey consisted of 56 statements and focused on assessing caretakers’ positive and negative attitudes (Table 1). Within the positive attitudes section, seven categories were addressed (Empathy affect; Empathy attribution; Comfortable with euthanasia; Confidence; Relying on others; Seek knowledge; and Use different sources to obtain advice) and included such statements as “When I see an unhappy pig, it upsets me more than it would upset most people” (Empathy affect) and “When I see a sick pig, I usually know if it will get better” [Confidence; (14)]. In addition, negative attitudes were also assessed, including five categories (Negative attitudes towards euthanasia; Insufficient knowledge; Negative attitudes about pigs; Perceived time constrains; and Trouble deciding when to euthanize and avoiding if possible) and statements as “I always try to save the pig before choosing to euthanize it” (Negative attitudes towards euthanasia) and “I do not have enough knowledge and/or experience to know when the pig needs to be euthanized” [Insufficient knowledge; (14)]. For each statement, individuals reported their responses on a 5-point Likert scale: (1) strongly disagree, (2) disagree, (3) neither agree nor disagree, (4) agree to (5) strongly agree. An additional “Choose not to disclose” option was also available. To ensure that statements were answered carefully and to mitigate bias and validate answers, four statements were reverse worded to contain a negation. The Likert scale for these specific questions was reversed with the higher agreement corresponding to a higher score for analysis.

**Table 1:** Second section of the survey: Survey statements attitudes^1^.

Attitude	Survey statement
Positive attitudes	
Empathy affect	Imagine how a pig feel is something I do often.
	I try to understand pigs by imagining how things looks like from their point of view.
	When I see pigs having fun, I feel really happy.
	If I see a pig injuring itself, I know how it feels.
	When I see an unhappy pig, it upsets me more than it would upset most people.
	I am better at telling if a pig is happy than most other people.
Empathy attribution	Pigs are generally able to feel sadness.
	I think pigs are generally able to feel happiness.
	Pigs have feelings like people have feelings.
	Pigs are sociable creatures.
	I consider that each pig is an individual with its own personality.
Comfortable with euthanasia	I feel comfortable euthanizing a pig.
Confidence	I am confident that I know when a pig needs to be euthanized.
	When I see a sick pig, I usually know if it will get better.
	When I see a sick pig, I usually know what is wrong with it.
	It is easy to identify a sick pig in the farm’s routine.
Relying on others	I can rely on my co-workers to monitor sick pigs when I am away from work.
	My coworkers are as good as I am at identifying and caring for sick pigs.
	I do not like to depend on other people to take care of sick pigs that are in my care (R)^2^.
Seek knowledge	I regularly check work instructions for how to deal with sick pigs.
	The farm veterinarian regularly gives us instructions on how to handle sick pigs.
	I update my knowledge of handling sick pigs regularly.
Use different sources to obtain advice	On the farm where I work, there are instructions on how to deal with sick pigs.
	I use the internet to help me diagnose what is wrong with a sick pig.
	My supervisor helps me diagnose what is wrong with a sick pig.
	The farm veterinarian helps me diagnose what is wrong with a sick pig.
	I use written references and notes to help me identify what is wrong with a sick pig.
	I rely on my co-workers to help me identify what is wrong with a sick pig.
	I ask co-workers for advice on how to diagnose a sick pig.
Negative attitudes	
Negative attitudes towards euthanasia	I always try to save the pig before choosing to euthanize it.
	If I could choose, I would prefer someone else to euthanize the pig.
	I do not like to perform the euthanasia procedure on pigs.
	I try to save all adult pigs, even if it takes a few days.
	I try to save all piglets before choosing to euthanize them when necessary.
	I try not to think about the pig’s feelings when I euthanize.
Insufficient knowledge	I do not have enough knowledge and/or experience to know what to do with a sick or compromised pig.
	I do not have enough knowledge and/or experience to know when the pig needs to be euthanized.
	I do not have enough knowledge and/or experience to diagnose what is wrong with sick pigs.
Negative attitudes about pigs	Seeing a neglected animal does not affect me as much as it would affect some people.
	Pigs are unfriendly.
	Pigs’ behavior it is not affected by the way we treat them.
	I think of pigs as generally being dirty.
Perceived time constrains	I am responsible for a large number of animals.
	I have a lot of sick pigs to take care of.
	The pigs are usually too crowded together for me to be able to inspect them carefully and properly.
	It is difficult to enter the pens to inspect the animals.
	During my working day, I perform other routine tasks before inspecting the pigs.
	I have enough time during my workday to identify sick pigs. (R)^2^
	I have as much time on weekends to inspect the pigs as I do on weekdays (R)^2^
Trouble deciding when to euthanize and avoiding if possible	It is difficult to decide when to euthanize a sick pig.
	I tend to wait longer than I should before euthanizing a pig.
	I often feel that there are good reasons not to euthanize the sick pig.
	I tend to disagree when a co-worker says a pig needs to be euthanized.
	I am more likely to euthanize a pig now than I was five years ago (R)^2^
	I am less likely to euthanize a sow that is close to farrowing than other sows.
	I know that euthanasia is the right thing to do to stop the pig from suffering, but I feel bad about having to do the procedure.

1 Responses were reported on a 5-point Likert Scale, from (1) strongly disagree, (2) disagree, (3) neither agree nor disagree, (4) agree to (5) strongly agree. (R)^2^ The scale was later reversed for analyses with higher score corresponding to higher agreement.

The final section of the survey, consisting of seven questions, was divided into two parts: knowledge about the existing material and guidelines for swine euthanasia in Brazil (7) and preferred methods of euthanasia. Respondents were asked to identify what they considered to be the most effective and appropriate euthanasia method for each stage of production (e.g., breeders, suckling piglets, nursery piglets, and finisher pigs). They were instructed to choose only one option for each category.

### 2.2 Statistical analyses

Cluster, univariate, and multivariable analyses were conducted using Stata/IC 17 (StataCorp., College Station, Texas). Data were initially checked for missing values and recording errors. Responses to statements and demographic questions that were left blank or in which participants opted not to disclose were considered missing data. Basic descriptive analyses, including descriptive plots and summary statistics (mean, standard deviation, range) were performed prior to conducting multivariable risk factor analyses.

Cluster analyses were employed to study participants in order to understand groupings with similar responses. The complete-linkage Cluster method was used with continuous dissimilarity measure and based on L2 or Euclidian distance. For this analysis, all variables from the survey section 2 (Table 1) were used.

Following this, a mixed-effect logistic multivariable model was developed, with the outcome variable of interest being Cluster membership (dichotomized as ‘yes’ or ‘no’). Predictors included demographical factors such as age, sex, racial identity, farm size, role at the farm, experience with pigs (in years), and previous and recent euthanasia experience. Previous euthanasia experience was defined as having ever euthanized an animal, regardless of whether it occurred before or after commencing work with swine. Recent euthanasia experience was defined as having euthanized an animal within the past 6 months.

The first step in model-building was to check for linearity between continuous variables and the log odds of the outcome. Since this assumption was not met for all variables, those were categorized as follows: pig experience was classified into three categories (0: ≤ 2 years of experience; 1: >2 to <10 years of experience; and 2: ≥ 10 years of experience); age was categorized into two groups (0: ≤ 36 years and 1: >36 years); racial identity was divided up into three categories (1: white; 2: black; and 3: brown); farm size was categorized into two groups (1: 1,001–2,000 housed sows; and 2: 2,001–3,500 housed sows); and role on the farm was classified into three categories (0: others [e.g., farm manager, animal scientists, veterinarians]; 1: department head; 2: caretakers).

Univariable mixed-effects logistic regression models were built for each predictor individually, using a *p* value <0.2 for screening predictors for inclusion in the final model. Multicollinearity among variables selected for the final model was assessed using the Spearman correlation coefficient, with a cut-off of 0.80. Multivariable mixed-effects models were then built using a backward stepwise approach, with statistical significance set at *p* < 0.05 and a tendency towards significance considered at 0.05 ≤ *p* < 0.10.

## 3 Results

A total of 117 individuals participated in this study. The farms varied in size, housing between 1,000 to 3,500 sows, with an annual finishing inventory target of 29,000 and 102,000 market hogs.

### 3.1 Section one—Demographics

Descriptive statistics on demographic information and euthanasia experience of the study participants are presented in Table 2.

**Table 2:** Descriptive analysis on demographics information and euthanasia experience from study participants.

Variable categorization	Value
*Continuous variables*	*Years*
Age	
Mean	36
Median	34
SD^1^	12.56
Range	18-65
Pig experience	
Mean	9.0
Median	5.0
SD	9.34
Range	0.01–40.00
*Categorial variables*	*% (n)*
Sex, % (*n*)	
Male	82.91 (97)
Female	17.09 (20)
Racial identity, % (*n*)^2^	
White	42.74 (50)
Brown	46.15 (54)
Black	10.26 (12)
Choose not to disclose	0.85 (1)
Place growing up, % (*n*)	
Big cities	8.55 (10)
Inner cities	41.03 (48)
Rural	49.57 (58)
Choose not to disclose	0.85 (1)
Highest degree or level of education, % (*n*)	
No formal education	2.56 (3)
Early childhood education	11.97 (14)
Primary education	28.21 (33)
Secondary education	40.17 (47)
Higher education	11.97 (14)
Postgraduate education	4.27 (5)
Choose not to disclose	0.85 (1)
Role on farm, % (*n*)	
Department head	16.24 (19)
Caretaker	70.94 (83)
Others	12.82 (15)
Working sector, % (*n*)	
Breeding	21.37 (25)
Farrowing	30.77 (36)
Nursery	11.97 (14)
Grow/finish	16.24 (19)
Wean-to-finish	7.69 (9)
All phases	11.11 (13)
Choose not to disclose	0.85 (1)
Sow inventory, number of sows housed	
From 1,001 to 2,000	51.28 (60)
From 2,001 to 3,500	48.72 (57)
Previous experience with other species, % (*n*)	
Yes	48.72 (57)
No	50.43 (59)
Choose not to disclose	0.85 (1)
Euthanasia experience with other species, % (*n*)	
Yes	69.23 (81)
No	29.06 (34)
Choose not to disclose	1.71 (2)
First euthanasia, % (*n*)	
Before I started working with pigs	16.24 (19)
After I started working with pigs	53.85 (63)
I have never euthanized an animal	21.37 (25)
Choose not to disclose	8.55 (10)
Performed euthanasia in the last 6 months, % (*n*)	
Yes	44.44 (52)
No	42.74 (50)
I have never euthanized a pig	11.11 (13)
Choose not to disclose	1.71 (2)
Previous training of pig euthanasia, % (*n*)	
Yes	36.75 (43)
No	54.70 (64)
Choose not to disclose	8.55 (10)

1 SD: Standard deviation.

2 In Brazil, racial identity is self-assessed and can be divided into the following main categories: *Branco* (White); *Pardo* (Brown—a range of mixed-race identities); *Preto* (Black); *Amarelo* (Yellow—Asian descent); *Indígena* (Indigenous)—Censo Demográfico, IBGE, 2022.

### 3.2 Section two—Positive and negative attitudes towards euthanasia

#### 3.2.1 Cluster analysis

**Figure 1:**
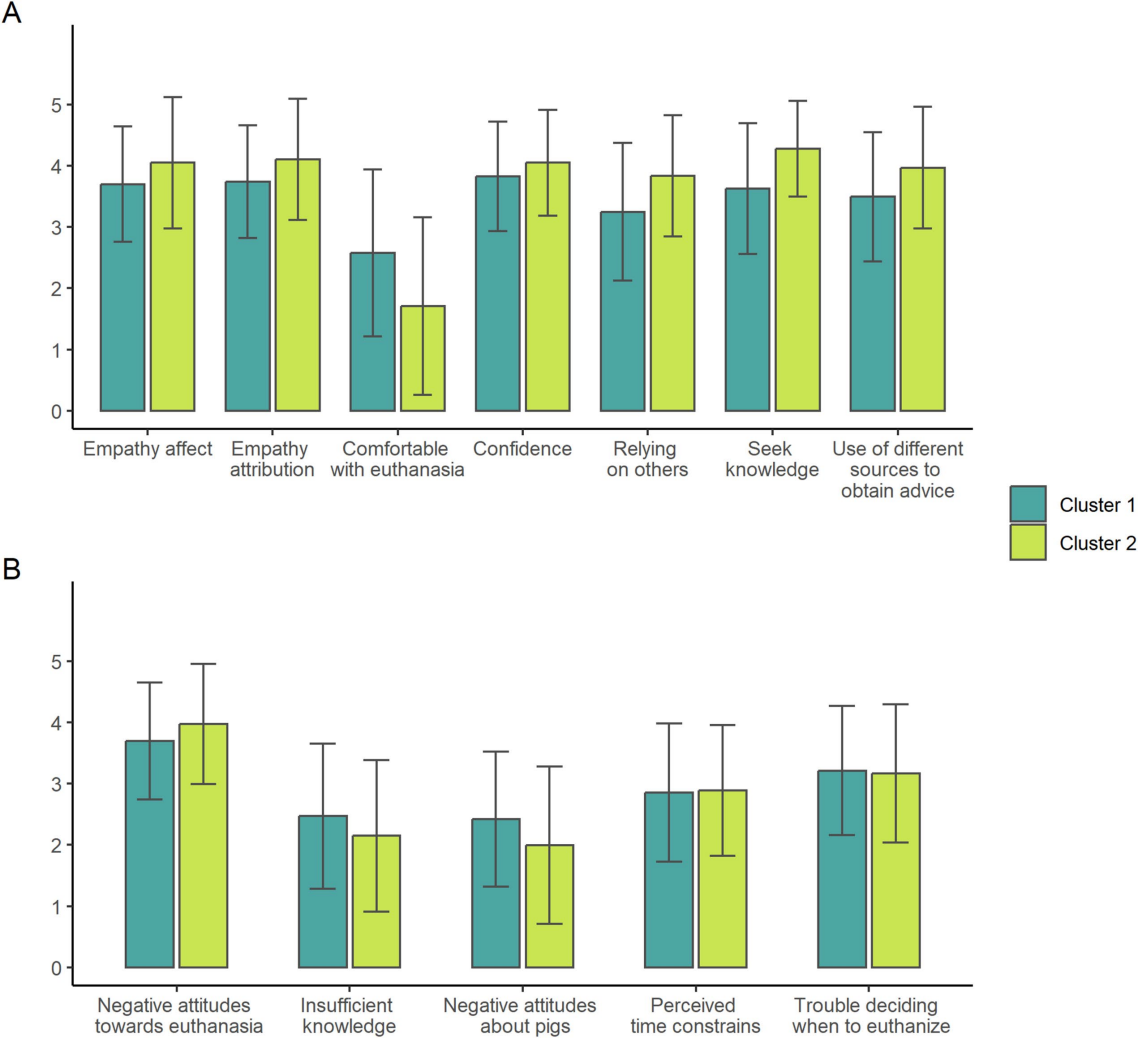
Cluster’s positive **(A)** and negative **(B)** attitudes according to combined survey statements (mean + SD) on a 5-point Likert Scale. Cluster 1: empathetic, self-sufficient, apathetic about euthanasia; and Cluster 2: empathic, knowledge seeker, uncomfortable with euthanasia. We generate the figure on R, using research data.

Cluster analysis identified two significantly different Clusters (Figure 1). Detailed information and values for each of the 56 statements are available in Supplementary Table S1.

##### 3.2.1.1 Cluster 1—empathetic, self-sufficient, apathetic about euthanasia

Cluster 1 consisted of 59 participants (50.43% of total). Participants in this Cluster displayed high scores for empathy and understanding toward pigs (Figure 1A), believing that pigs can experience emotions such as sadness and happiness (e.g., “Empathy attributions,” Table 1). They recognized pigs’ individuality and sociability (e.g., “Empathy attributions,” Table 1). and had a neutral to slightly positive view of pigs’ cleanliness (e.g., “Negative attitudes about pigs,” Table 1). Members of Cluster 1 displayed high scores in confidence in diagnosing and managing sick pigs (Confidence, Figure 1A) and did not overly depend on seeking out help or knowledge from co-workers or other resources (e.g., “Relying on others” and “Seek knowledge”; Table 1). They demonstrated a strong sense of responsibility towards treating pigs and were committed to saving pigs before considering euthanasia (e.g., “Negative attitudes towards euthanasia,” Table 1). In cases where treatment was unsuccessful and euthanasia was required, members felt apathetic towards making the decision to euthanize (Comfortable with euthanasia, Figure 1A) and performing the procedure itself (Trouble deciding when to euthanize, Figure 1B). Despite this apathy, participants indicated that euthanasia was not delayed if and when necessary, and when performed, individuals tried not to focus on the pig’s feelings during the process (e.g., “Trouble deciding when to euthanize,” Table 1).

##### 3.2.1.2 Cluster 2—empathetic, knowledge seeker, uncomfortable with euthanasia

Cluster 2 consisted of 58 participants (49.57% of total). Members of this Cluster exhibited a strong level of empathy and understanding towards pigs (Figure 1A), strongly believing in pigs’ emotional capabilities and individuality (e.g., “Empathy affect” and “Empathy attribution,” Table 1). They were sensitive to pigs’ emotions and more affected to the pig’s condition when compared to Cluster 1 (Empathy affect and attribution, Figure 1A). Cluster 2 participants were confident in diagnosing and managing sick pigs (e.g., “Confidence,” Table 1), but in contrast to Cluster 1, Cluster 2 members relied on co-workers and other references to make decisions (e.g., “Relying on others” and “Seek knowledge,” Table 1). Cluster 2 members were committed to saving pigs (e.g., “Negative attitudes towards euthanasia”) but felt uncomfortable making the decision to euthanize and perform the procedure (Comfortable with euthanasia, Figure 1A). Despite this discomfort, Cluster 2 members did not feel that euthanasia was delayed if needed (e.g., “Trouble deciding when to euthanize and avoiding if possible”), and as demonstrated in Cluster 1 responses, Cluster 2 members also tried not to focus on the pig’s feelings when performing euthanasia (e.g., Negative attitudes towards euthanasia, Figure 1B).

#### 3.2.2 Risk factor analysis

Table 3 presents the final model for the risk factor analysis. Farm size and age were the only variables that showed a tendency (0.05 ≤ *p* < 0.10) to be associate with Cluster membership. Participants working on large-sized farms (2,000 to 3,500 sows) and older individuals (>36 years old) had 2.09 and 2.07 times the odds, respectively, of being in Cluster 1 compared to Cluster 2.

**Table 3:** Final risk analysis model for Cluster 1.

Variable	Category	OR^1^	SE^2^	95% CI^3^	*p*-*value*
Cluster 1: empathetic, self-sufficient, apathetic about euthanasia					
Farm size					
1,000 to 2,000 sows		Referent			
2,001 to 3,500 sows		2.094	0.795	0.995, 4.405	0.051
Age					
≤ 36 years		Referent			
> 36 years		2.077	0.783	0.992, 4.347	0.052

1 Odds ratio.

2 Standard error.

3 Comfidence interval.

### 3.3 Section three—Previous knowledge regarding euthanasia and methods

Table 4 presents the results from the third section of the survey, in which respondents selected the most effective and appropriate euthanasia method for all stages of production and if they were aware of the on-farm swine euthanasia guidelines developed by Ministry of Agriculture, Livestock and Food Supply (7).

**Table 4:** Caretakers’ knowledge regarding euthanasia method and resources available.

Euthanasia knowledge	
Variable	Number, % (*n*)
Are you aware of the recommendations of the MAPA^1^ regarding the criteria for performing euthanasia on pig farms?	
Yes	32.48 (38)
No	56.41 (66)
Choose not to disclose	11.11 (13)
Have you read and/or studied the booklet “Euthanasia of Pigs on Farms”?	
Yes	9.40 (11)
No	83.76 (98)
Choose not to disclose	6.84 (8)

Methods preferences		
Category	Preferred method	Number, % (*n*)
Breeders (sows and boars)	Electrical stunning	43.59 (51)
	Penetrating captive bolt	43.59 (51)
	Concussion (Sledgehammer)	4.27 (5)
	Cardiac Perforation	4.27 (5)
	Anesthetic overdose	0.85 (1)
	Choose not to disclose	3.42 (4)
Suckling piglets	Non-penetrating bolt	42.74 (50)
	Manual blunt force trauma + bleeding	24.79 (29)
	Manual blunt force trauma	18.18 (22)
	Anesthetic overdose	3.42 (4)
	Electrical stunning	2.56 (3)
	Cardiac Perforation	0.85 (1)
	Choose not to disclose	6.84 (8)
Nursery piglets (> 10 kg)	Electrical stunning	31.62 (37)
	Penetrating captive bolt	26.50 (31)
	Non-penetrating bolt	20.51 (24)
	Manual blunt force trauma + bleeding	11.11 (13)
	Manual blunt force trauma	0.85 (1)
	Anesthetic overdose	0.85 (1)
	Cardiac perforation	0.85 (1)
	Choose not to disclose	7.69 (9)
Growing-finishing pigs	Penetrating captive bolt	34.19 (40)
	Electrical stunning	32.48 (38)
	Non-penetrating bolt	13.68 (16)
	Cardiac perforation	6.84 (8)
	Concussion (Sledghammer)	2.56 (3)
	Anesthetic overdose	0.85 (1)
	Choose not to disclose	9.40 (11)
Exsanguination^2^	Yes	22.22 (26)
	No	64.10 (75)
	Choose not to disclose	13.68 (16)

1 MAPA: ‘*Ministério da Agricultura, Pecuária e Abastecimento’ -* Ministry of Agriculture, Livestock and Food Supply, Brazil.

2 The final question addressed whether the pig should be exsanguinated after the stunning, regardless of whether the method is reversible or irreversible.

## 4 Discussion

Timely and humane euthanasia is a critical aspect of animal welfare on swine farms (1, 2). It serves as a necessary intervention to alleviate the suffering of pigs with poor recovery prospects. In Brazil, while euthanasia is designated as a veterinarian’s responsibility, its practical execution often falls to swine farm caretakers. This reliance on caretakers may introduce some challenges given the majority of caretakers lack training and confidence in performing euthanasia (10). Understanding how caretaker knowledge, attitudes and perceptions influence euthanasia performance is needed. Therefore, the objective of this study was to assess Brazilian caretakers’ knowledge and attitudes towards on-farm swine euthanasia and identify barriers to implementing euthanasia in a timely manner.

In this study, most surveyed caretakers identified as male (82.9%), between 18 and 65 years old, with an average of 10 years’ pig experience (± 9.7, range: 2 days to 40 years). In contrast, female caretakers represented less than 20% of surveyed participants, were generally younger (Range: 21 to 51 years old, Avg: 30 years ±8.4) and had less experience (Avg: 4.5 ± 5.5 years) than their male counterparts. Results from this study mirror population demographics for on-farm caretakers across food animal sectors on a global scale (15) and are similar to populations of surveyed participants in previous work addressing caretakers’ attitudes toward euthanasia in the swine [58–86% male representation; (10, 13, 16, 17)], and dairy industry [78–80% male representation; (12, 18)].

Sex and age have been shown to influence perceptions of and confidence regarding euthanasia decision-making. Male caretakers often exhibit more apathy toward performing euthanasia compared to female caretakers (13, 18–20), while female caretakers tend to display higher levels of empathy and emotional connection towards pigs, which can increase the discomfort regarding euthanasia decisions (13). Similarly, age can affect both perceptions and willingness to perform euthanasia, with younger individuals generally expressing greater discomfort with on-farm euthanasia (13, 21). In this study, participants under 36 years of age tended to feel more uncomfortable (*p* = 0.052) about performing euthanasia compared to older participants and aligns with previous swine-specific euthanasia research (13).

Although this study represents only a small sample of the Brazilian swine industry, it highlights the importance of recognizing how caretaker demographics can influence euthanasia standards and expectations on-farm. Euthanasia training and on-farm implementation must adapt to accommodate changes in the demographic dynamics of those working with pigs, particularly considering shifting demographics in agriculture and the impeding retirements of experienced workers (22). One key challenge in swine euthanasia is the resistance and discomfort towards euthanasia from new caretakers in the field (3), further emphasizing the need for tailored training approaches and development of open dialogues on-farm to address such challenges.

In addition to caretaker demographics, farm-size can also influence the effective implementation of euthanasia on-farm. Previous work by Campler et al. (13) in swine and Merenda et al. (12) in cattle demonstrated that caretakers from smaller farms may have better knowledge or competencies on how to handle sick or injured animals compared to those working on larger farms. However, small-farm caretakers are more likely to report negative emotions toward euthanasia (18). In our study, caretakers working on smaller farms tended (*p* = 0.051) to feel confident about identifying a sick pig, but uncomfortable with the euthanasia procedure itself. These results agree with previous work conducted with dairy cows’ caretakers demonstrating farm size can impact attachment levels towards animals as well as knowledge and competency performing euthanasia. From the dairy perspective, caretakers working on larger farms were less confident, more detached from the animals, and have less knowledge about euthanasia (12). Given the Brazilian swine industry future trajectory will likely result in larger, more consolidated farm systems (23, 24), understanding barriers to timely euthanasia implementation in such larger and consolidate systems is needed.

In addition to demographic factors influencing euthanasia decision-making, personnel characteristics can also influence the decision-making and implementation of euthanasia on-farm. In the present study, two Clusters were identified among participants surveyed. Cluster 1 participants, defined as empathetic, self-sufficient yet apathetic about euthanasia, represented approximately half of the participating group. These individuals displayed strong empathy and understanding towards pigs, believing pigs are sentient beings that can experience different types of emotions. Cluster 1 participants were confident in identifying compromised animals requiring treatment and needed little to no assistance or guidance from others on-farm to make treatment decisions. However, despite the strong emotional connection to healthy pigs and knowledge pertinent to diagnosing and treating sick pigs, Cluster 1 members demonstrated apathy towards making the decision to euthanize and performing the procedure itself.

Cluster 1 characteristics are similar to previous euthanasia attitude work performed in swine, identifying subpopulations of caretakers that were confident with treating sick pigs yet detached emotionally from the euthanasia process (13, 25). Several factors may be attributing to this caretaker profile. First, individuals with extensive experience managing swine health and disease on-farm often use objective animal-based outcome measures as part of the decision-making process (25–27). This expertise enables them to better assess treatment options and predict the likelihood of recovery for an individual animal using minimal diagnostic testing (1, 35). For example, work conducted on piglet birth weight have shown that piglets born weighting less than 0.86 kg, coupled with impaired vitality scores, have a 94% probability of death by day five of age (28). Therefore, individuals with such knowledge may feel more confident in their ability to predict health outcomes and, consequently, can better safeguard the welfare of the animal by making quicker decisions. However, this confidence does not necessarily imply that euthanasia is performed less frequently. Rather, as caretakers gain more experience with common diseases and injuries encountered at different production stages, the euthanasia decision-making process becomes less emotionally taxing, as the evidence supporting whether a pig should be euthanized or treated is more apparent (15, 25).

Another factor that may be influencing the overall apathy towards euthanasia decision-making and performance in Cluster 1 is the frequency in which euthanasia is performed on farm. Over 40% of Cluster 1 participants stated they had not conducted euthanasia in the past 6 months, 15% had never performed euthanasia, and 37% had never received euthanasia training. This lack of experience may contribute to a reduced awareness of the euthanasia process, potentially diminishing emotional engagement and decision-making urgency, especially if euthanasia is not a regular part of their daily responsibilities. However, it is also possible that the lack of experience could contribute to stress and anxiety about performing euthanasia correctly, as some individuals may feel unprepared or uncertain about their ability to execute the procedure properly. Additionally, many participants may not have had the opportunity or authority to perform euthanasia, as most held caretaker roles, with larger systems often designating one or two employees to make euthanasia decisions and carry out the procedure (12). To better understand the apathy observed in Cluster 1, future research should explore caretakers’ perceptions of euthanasia in greater depth, such as through interviews and focus groups. This approach would provide a more nuanced understanding of the role euthanasia plays in their daily tasks and its emotional impact.

Cluster two participants represented the other half of the surveyed population and shared many similarities with Cluster 1, including a high level of empathy towards pigs and the belief that pigs are sentient beings. However, Cluster 2 participants differed in that they relied more on other resources (co-workers and material) to make euthanasia decisions and expressed emotional discomfort with the euthanasia process. As highlighted in previous work in both swine and dairy industries, the characteristics of Cluster 2 participants are similar to those of other profiles, with individuals demonstrating empathy while being uncomfortable with the decision-making and euthanasia performance process (12, 13). Caretakers’ attitudes towards pigs significantly influence their willingness to perform euthanasia (3, 16, 29), and caretakers often experience negative emotions associated with euthanasia, including guilt and stress related to performing the procedure (14, 30). Exposure to such emotional events can trigger individuals to delay or avoid performing or participating in euthanasia as a result of the “caring-killing paradox” [i.e., tension between providing care and making euthanasia decision; (4, 31, 32)].

In contrast to Cluster 1, the majority of Cluster 2 participants (72%) reported never receiving euthanasia training. Additionally, nearly half (48%) of Cluster 2 members had not performed euthanasia recently, and 9% had never conducted euthanasia at all. These findings underscore the need to further investigate how euthanasia is being implemented on-farm given a humane death for any suffering animal is a priority for the Brazilian swine industry. Thus, developing educational materials and resources to support the mental impact of performing euthanasia and ensuring euthanasia is an accepted tool that needs to be implemented on-farm to relieve animal suffering is critically important.

The last component of this study was to determine previous knowledge regarding euthanasia methods and protocols currently being implemented on-farm. When evaluating data by production stage, 8.5% of participants selected unacceptable methods (such as cardiac perforation and concussion) for breeding stock, while 19% chose blunt force trauma not followed by exsanguination (which is a requirement by MAPA) for pre-weaned piglets. Additionally, 23–33% of participants selected unacceptable methods (including non-penetrating captive bolt, blunt force trauma, and cardiac perforation) for nursery and finisher pigs. These findings reflect similar results to previous work looking at euthanasia methods, where two of the most commonly selected methods (electrocution with homemade equipment and cardiac perforation) are prohibited by legislation under any circumstances (10). The preference for cardiac perforation and electrocution with homemade equipment found in the Dalla Costa et al. (10) study is likely related not only to the minimal cost of these procedures compared to the use of recommended methods with appropriate equipment, but also to the visual aspect of the procedure. Caretakers may prefer these methods because the tonic and clonic convulsions are less intense and shorter in duration compared to the more pronounced motor responses seen with methods such as the captive bolt (10, 33). However, tonic and clonic convulsions are normal and indicate that the procedure was performed correctly (34), although caretakers may mistakenly perceive them as signs of consciousness (33).

Additionally, 64% of respondents, believed that exsanguination was unnecessary following insensibilization, indicating a failure in the second step of euthanasia process, according to Brazilian swine euthanasia guidelines (7). This, in conjunction with the emotional barriers associated with euthanasia, highlight the urgent need for significant work to ensure caretakers can not only confidently identify compromised pigs and determine when to implement euthanasia and consistently utilize appropriate and approved tools for euthanasia by production stage. Collaboration with MAPA and state/country organizations will be an important starting point to ensure euthanasia is implemented on-farm in a timely and humane manner.

## 5 Conclusion

The findings from this study provide valuable insights into the experiences, knowledge, and attitudes of swine caretakers regarding euthanasia practices. Caretakers generally demonstrate strong empathy for pigs, acknowledging their capacity to experience pain and suffering. However, notable differences emerged across demographics and farm contexts. Younger caretakers and those on smaller farms reported greater discomfort with performing euthanasia, often linked to emotional challenges and limited technical confidence. Additionally, some caretakers stated the use of non-appropriate euthanasia techniques for different categories of pigs, raising significant concerns about both animal welfare and adherence to recommended practices. Furthermore, a substantial number of caretakers did not consider the second step of the euthanasia process, such as exsanguination, to be important. This oversight highlights critical gaps in knowledge and practice, reinforcing the need for targeted training programs to ensure adherence to proper euthanasia procedures.

Key barriers identified include unclear euthanasia protocols, inconsistent knowledge of legislative requirements, and the psychological toll associated with performing euthanasia. These challenges adversely impact caretakers’ confidence and comfort levels, often delaying timely and humane decision-making. Addressing these issues is essential to advancing animal welfare standards and equipping caretakers with the tools and knowledge needed to manage the complexities of the euthanasia process effectively. As part of a broader project, this study provides the foundation for developing a comprehensive training tool tailored to the unique challenges faced by swine caretakers. This tool will emphasize practical skill development for humane euthanasia techniques, including the critical importance of performing all procedural steps, such as exsanguination. Moreover, it will enhance caretakers’ awareness of legislative and welfare requirements while integrating strategies to build emotional resilience to cope with the psychological demands of the process.

Future efforts should prioritize collaboration with industry stakeholders, policymakers, and animal welfare experts to ensure the tool’s relevance and practicality across diverse farming contexts. Field evaluations and ongoing feedback from caretakers will be crucial for refining the tool and ensuring its adaptability to real-world conditions. By addressing the gaps identified in this study, these initiatives have the potential to empower caretakers to make informed, timely, and humane decisions, reducing unnecessary animal suffering and promoting higher welfare standards on swine farms.

## Data Availability

The original contributions presented in the study are included in the article/Supplementary material, further inquiries can be directed to the corresponding author.
