# Supplementary material for: Perceptions of swine euthanasia among Brazilian caretakers from non-integrated swine farms
Source: Front Vet Sci. 2025 Jan 6;11:1513141. doi: 10.3389/fvets.2024.1513141 (PMC11743181; doi:10.3389/fvets.2024.1513141)
Supplement: Supplementary file 1 [file Table_1.DOCX]

Supplementary Material

# Appendix 1 – Survey section 1 – demographic data of caretakers

**Section 1 – You and Swine Production**

In this section, we aim to understand your personal background and your relationship with animal production.

1. **What is your age?** __________________________________________________
2. **What is your gender? (Please select one option)**
   - Male
   - Female
   - Non-binary
   - Other _______________________________________________________
   - Choose not to disclose
3. **Please specify your racial identity. (Please select one option)**
   - Black
   - Brown
   - White
   - Yellow (e.g., Asian)
   - Indigenous
   - Choose not to disclose
4. **Where did you spend most of your time while growing up? (Please select one option)**

- Big cities
- Inner cities
- Rural
- Choose not to disclose

1. **What is the highest degree or level of education you have received? (Please select one option)**
   - No Formal Education
   - Early Childhood Education
   - Primary Education
   - Secondary Education
   - Higher Education
   - Postgraduate Education
   - Choose not to disclose
2. **How long have you worked with pigs?**

- Months _____________________________________________________
- Years ______________________________________________________
- Choose not to disclose

1. **How would you describe your current role on the swine farm? (Please select one option)**
   - Farm manager
   - Department head
   - Caretaker
   - Other ______________________________________________________
   - Choose not to disclose
2. **Where on the swine farm do you spend most of your time? (Please select one option)**

- Replacement and breeding
- Farrowing
- Nursery
- Grow/finish
- Wean-to-finish
- Quarantine
- All production sites
- Choose not to disclose

1. **What size of farm do you work on? (Please select one option)**

- Less than 1,000 housed sows
- From 1,001 to 2,000 housed sows
- From 2,001 to 3,500 housed sows
- From 3,501 to 5,000 housed sows
- More than 5,000 housed sows
- Choose not to disclose

1. **Did you work with other livestock species before working with pigs? (Please select one option)**

- Yes
- No
- Choose not to disclose

1. **If so, what other livestock species have you worked with? ________________**
2. **Have you ever euthanized a livestock species?**

- Yes
- No
- Choose not to disclose

1. **When was the first time you had to euthanize a livestock animal?**

- Before I started working with pigs
- After I started working with pigs
- I have never euthanized an animal
- Choose not to disclose

1. **Have you euthanized an animal in the last 6 months?**

- Yes
- No
- I have never euthanized a pig
- Choose not to disclose

1. **Have you ever participated in any technical training on the euthanasia of pigs on farms?**

- Yes
- No
- Choose not to disclose

# Supplementary Table

| **Supplementary Table 1.** Cluster agreement to survey statement (average ± SD). | | |
| --- | --- | --- |
| **Survey Statement** | **Average response^1^** | |
|  | **Cluster 1** | **Cluster 2** |
| ***Positive attitudes*** |  |  |
| *Empathy affect* | 3.701 ± 0.297 | 4.049 ± 0.357 |
| Imagine how a pig feel is something I do often. | 3.796 ± 0.924 | 4.138 ± 1.206 |
| I try to understand pigs by imagining how things looks like from their point of view. | 3.424 ± 0.913 | 4.241 ± 0.979 |
| When I see pigs having fun, I feel really happy. | 4.152 ± 0.887 | 4.465 ± 0.883 |
| If I see a pig injuring itself, I know how it feels. | 3.898 ± 0.845 | 4.224 ± 1.044 |
| When I see an unhappy pig, it upsets me more than it would upset most people. | 3.441 ± 1.055 | 3.672 ± 1.289 |
| I am better at telling if a pig is happy than most other people. | 3.491 ± 1.040 | 3.552 ± 1.029 |
| *Empathy attribution* | 3.739 ± 0.210 | 4.103 ± 0.266 |
| Pigs are generally able to feel sadness. | 3.729 ± 0.944 | 4.293 ± 0.918 |
| I think pigs are generally able to feel happiness. | 4.068 ± 0.640 | 4.465 ± 0.681 |
| Pigs have feelings like people have feelings. | 3.491 ± 1.073 | 3.931 ± 1.212 |
| Pigs are sociable creatures. | 3.661 ± 0.976 | 4.000 ± 0.991 |
| I consider that each pig is an individual with its own personality. | 3.746 ± 0.956 | 3.827 ± 1.141 |
| *Comfortable with euthanasia* | 2.576 ± 0.000 | 1.707 ± 0.000 |
| I feel comfortable euthanizing a pig. | 2.576 ± 1.367 | 1.707 ± 1.451 |
| *Confidence* | 3.826 ± 0.160 | 4.052 ± 0.318 |
| I am confident that I know when a pig needs to be euthanized. | 3.830 ± 0.985 | 3.707 ± 1.298 |
| When I see a sick pig, I usually know if it will get better. | 3.729 ± 0.806 | 4.138 ± 0.605 |
| When I see a sick pig, I usually know what is wrong with it. | 3.694 ± 0.914 | 3.914 ± 0.822 |
| It is easy to identify a sick pig in the farm’s routine. | 4.051 ± 0.859 | 4.448 ± 0.730 |
| *Relying on others* | 3.469 ± 0.561 | 3.592 ± 1.268 |
| I can rely on my co-workers to monitor sick pigs when I am away from work. | 3.881 ± 0.948 | 4.465 ± 0.706 |
| My coworkers are as good as I am at identifying and caring for sick pigs. | 3.695 ± 1.118 | 4.172 ± 0.881 |
| I do not like to depend on other people to take care of sick pigs that are in my care (R)^2^. | 2.830 ± 1.302 | 2.138 ± 1.382 |
| *Seek Knowledge* | 3.627 ± 074 | 4.276 ± 0.096 |
| I regularly check work instructions for how to deal with sick pigs. | 3.542 ± 1.164 | 4.190 ± 0.805 |
| The farm veterinarian regularly gives us instructions on how to handle sick pigs. | 3.678 ± 1.074 | 4.259 ± 0.870 |
| I update my knowledge of handling sick pigs regularly. | 3.661 ± 0.958 | 4.379 ± 0.671 |
| *Use different sources to obtain advice* | 3.496 ± 0.548 | 3.968 ± 0.575 |
| On the farm where I work, there are instructions on how to deal with sick pigs. | 3.813 ± 0.955 | 4.052 ± 1.176 |
| I use the internet to help me diagnose what is wrong with a sick pig. | 2.322 ± 1.074 | 2.793 ± 1.484 |
| My supervisor helps me diagnose what is wrong with a sick pig. | 3.949 ± 1.024 | 4.345 ± 0.807 |
| The farm veterinarian helps me diagnose what is wrong with a sick pig. | 3.576 ± 1.102 | 4.086 ± 0.996 |
| I use written references and notes to help me identify what is wrong with a sick pig. | 3.424 ± 1.133 | 3.707 ± 1.185 |
| I rely on my co-workers to help me identify what is wrong with a sick pig. | 3.813 ± 1.008 | 4.448 ± 0.653 |
| I ask co-workers for advice on how to diagnose a sick pig. | 3.576 ± 1.086 | 4.345 ± 0.664 |
| ***Negative attitudes*** | | |
| *Negative attitudes towards euthanasia* | 3.698 ± 0.268 | 3.974 ± 0.218 |
| I always try to save the pig before choosing to euthanize it. | 3.966 ± 0.909 | 4.259 ± 1.236 |
| If I could choose, I would prefer someone else to euthanize the pig. | 3.441 ± 1.178 | 3.879 ± 1.229 |
| I do not like to perform the euthanasia procedure on pigs. | 3.424 ± 1.133 | 4.00 ± 1.0429 |
| I try to save all adult pigs, even if it takes a few days. | 3.983 ± 0.547 | 4.121 ± 0.462 |
| I try to save all piglets before choosing to euthanize them when necessary. | 3.864 ± 0.937 | 3.965 ± 1.042 |
| I try not to think about the pig's feelings when I euthanize. | 3.508 ± 1.023 | 3.621 ± 0.875 |
| *Insufficient knowledge* | 2.469 ± 0.125 | 2.149 ± 0.156 |
| I do not have enough knowledge and/or experience to know what to do with a sick or compromised pig. | 2.373 ± 1.216 | 2.086 ± 1.218 |
| I do not have enough knowledge and/or experience to know when the pig needs to be euthanized. | 2.424 ± 1.192 | 2.327 ± 1.369 |
| I do not have enough knowledge and/or experience to diagnose what is wrong with sick pigs. | 2.610 ± 1.145 | 2.034 ± 1.123 |
| *Negative attitudes about pigs* | 2.424 ± 0.380 | 1.996 ± 0.199 |
| Seeing a neglected animal does not affect me as much as it would affect some people. | 2.797 ± 1.310 | 2.086 ± 1.315 |
| Pigs are unfriendly. | 2.237 ± 1.023 | 2.224 ± 1.377 |
| Pigs' behavior it is not affected by the way we treat them. | 1.983 ± 0.956 | 1.776 ± 1.402 |
| I think of pigs as generally being dirty. | 2.678 ± 1.121 | 1.896 ± 1.038 |
| *Perceived time constrains* | 2.855 ± 0.658 | 2.892 ± 0.996 |
| I am responsible for a large number of animals. | 3.797 ± 1.095 | 4.103 ± 0.949 |
| I have a lot of sick pigs to take care of. | 2.186 ± 1.058 | 2.000 ± 0.878 |
| The pigs are usually too crowded together for me to be able to inspect them carefully and properly. | 2.898 ± 1.155 | 2.121 ± 0.938 |
| It is difficult to enter the pens to inspect the animals. | 2.119 ± 1.068 | 1.741 ± 1.117 |
| During my working day, I perform other routine tasks before inspecting the pigs. | 2.797 ± 1.256 | 3.069 ± 1.437 |
| I have enough time during my workday to identify sick pigs. (R)^2^ | 3.644 ± 0.978 | 4.207 ± 0.894 |
| I have as much time on weekends to inspect the pigs as I do on weekdays (R)^2^ | 2.542 ± 1.264 | 3.000 ± 1.256 |
| *Trouble deciding when to euthanize and avoiding if possible* | 3.213 ± 0.135 | 3.165 ±0.447 |
| It is difficult to decide when to euthanize a sick pig. | 3.051 ± 1.166 | 3.224 ± 1.377 |
| I tend to wait longer than I should before euthanizing a pig. | 3.305 ± 1.004 | 2.879 ± 1.125 |
| I often feel that there are good reasons not to euthanize the sick pig. | 3.322 ± 0.918 | 3.172 ± 1.028 |
| I tend to disagree when a co-worker says a pig needs to be euthanized. | 2.661 ± 0.976 | 2.362 ± 1.180 |
| I am more likely to euthanize a pig now than I was five years ago (R)^2^ | 3.390 ± 1.034 | 3.293 ± 1.108 |
| I am less likely to euthanize a sow that is close to farrowing than other sows. | 3.448 ± 1.259 | 3.448 ± 1.259 |
| I know that euthanasia is the right thing to do to stop the pig from suffering, but I feel bad about having to do the procedure. | 3.102 ± 1.213 | 3.776 ± 0.839 |
| ^1^Data are presented as the mean ± standard deviation. Responses were recorded on a 5-point Likert scale, ranging from 1) strongly disagree, 2) disagree, 3) neither agree nor disagree, 4) agree, to 5) strongly agree. ^2^The final question addressed whether the pig should be exsanguinated after the stunning, regardless of whether the method is reversible or irreversible. | | |
